# Effect of the Antihypertensive Drug Enalapril on Oxidative Stress Markers and Antioxidant Enzymes in Kidney of Spontaneously Hypertensive Rat

**DOI:** 10.1155/2014/608512

**Published:** 2014-08-28

**Authors:** G. Chandran, K. N. S. Sirajudeen, Nik Syamimi Nik Yusoff, M. Swamy, Mutum S. Samarendra

**Affiliations:** ^1^Department of Chemical Pathology, School of Medical Sciences, Universiti Sains Malaysia, Health Campus, 16150 Kubang Kerian, Kelantan, Malaysia; ^2^Department of Pathology, School of Medical Sciences, Universiti Sains Malaysia, Health Campus, 16150 Kubang Kerian, Kelantan, Malaysia

## Abstract

Oxidative stress has been suggested to play a role in hypertension and hypertension induced organ damage. This study examined the effect of enalapril, an antihypertensive drug, on oxidative stress markers and antioxidant enzymes in kidney of spontaneously hypertensive rat (SHR) and N*ω* -nitro-L-arginine methyl ester (L-NAME) administered SHR. Male rats were divided into four groups (SHR, SHR+enalapril, SHR+L-NAME, and SHR+enalapril+L-NAME). Enalapril (30 mg kg^−1^ day^−1^) was administered from week 4 to week 28 and L-NAME (25 mg kg^−1^ day^−1^) was administered from week 16 to week 28 in drinking water. Systolic blood pressure (SBP) was measured during the experimental period. At the end of experimental periods, rats were sacrificed; urine, blood, and kidneys were collected for the assessment of creatinine clearance, total protein, total antioxidant status (TAS), thiobarbituric acid reactive substances (TBARS), superoxide dismutase (SOD), and catalase (CAT), as well as histopathological examination. Enalapril treatment significantly enhanced the renal TAS level (*P* < 0.001) and SOD activity (*P* < 0.001), reduced the TBARS levels (*P* < 0.001), and also prevented the renal dysfunction and histopathological changes. The results indicate that, besides its hypotensive and renoprotective effects, enalapril treatment also diminishes oxidative stress in the kidneys of both the SHR and SHR+L-NAME groups.

## 1. Introduction

Hypertension is a global chronic health condition in which systemic arterial pressure is persistently elevated. It is of great public concern as prolonged, uncontrolled hypertension leads to cardiovascular diseases and organ damage including the kidneys, resulting in nephropathy, chronic renal disease, and ultimately renal failure [1]. This makes it the leading behavioural and physiological risk factor for attributable deaths, accounting for 13% of global deaths [2].

The pathogenesis of essential hypertension is multifactorial and highly complex as various factors modulate the blood pressure in the body [3]. In this respect, free radical mediated oxidative damage has been proposed as an important predisposing pathogenic mechanism in the development and progression of hypertension and its complications including organ damage [4, 5]. Free radicals and their metabolites, reactive oxygen species (ROS), are constantly formed in the body by several mechanisms. These substances, being reactive, can cause oxidative damage to biological molecules. The body possesses antioxidant systems that are very important to protect cellular components from free radical induced oxidative damage. These consist of nonenzymatic and enzymatic systems including SOD and CAT [6]. Under physiological conditions, ROS produced in the course of metabolism are contained by the body's antioxidant defence mechanism. When these defence mechanisms are inadequate, either due to increased ROS production or diminished antioxidant levels, oxidative stress occurs [7]. Oxidative stress which leads to damage of biological molecules, such as lipids, proteins, carbohydrates, and DNA, can inflict tissue injury and dysfunction [8]. Several reports have documented that hypertension is associated with increased free radical production as well as reduction of antioxidant capacity [9, 10]. High levels of lipid peroxidation biomarkers [11, 12] as well as hydrogen peroxide [13] in patients with essential hypertension suggest the probable involvement of free radicals in this disease and its long term complications.

As hypertension contributes to organ damage, antihypertensive drug treatment aims to reduce blood pressure and hypertension induced organ damage including the kidneys. In this respect, studies have shown that certain antihypertensive drugs, in particular those that target the renin-angiotensin system, are able to blunt the progression of renal disease in hypertension [14–17]. Some studies have suggested that the therapeutic benefit of antihypertensive drugs including renoprotection could be in part due to their antioxidant properties whereby there is inhibition of free radical production. These studies involving both human and animal models, including the SHR, have demonstrated that certain groups of antihypertensive drugs, such as the angiotensin converting enzyme inhibitors (ACEi), angiotensin receptor blockers (ARB), and calcium channel blockers (CCB), lower blood pressure and cause changes in the oxidative status [18–22].

Even though antihypertensive drug treatments have been shown to reduce blood pressure and certain oxidative stress parameters, the studies concerned were not comprehensive as no in-depth study on the effect of these antihypertensive drug treatments on the antioxidant mechanisms involved as kidney damage progresses has been carried out. As such, the biochemical mechanisms by which these antihypertensive drugs might inhibit oxidative stress, especially in the kidneys, are not well known. Further studies are needed to clarify whether these antihypertensive drugs function by affecting the antioxidant defence mechanisms in the kidneys or just primarily correct the altered mechanical forces that cause structural changes in the kidney.

The SHR is a suitable model for the study of essential hypertension as the natural progression of hypertension and organ damage including the kidneys is remarkably similar to man. As in humans, kidney damage and progressive decline in glomerular filtration rate (GFR) occur at a much later stage in the SHR. Time-course studies until this stage of renal damage require maintaining SHR until an advanced age which would take a very long time and is costly. This is overcome by the usage of the L-NAME administered SHR model which produces renal damage similar to those seen in human hypertensive nephropathy [23]. This model has been used for studies on hypertensive nephropathy [24–26].

Overall the effect of ACEi in lowering blood pressure on oxidative stress parameters and related protective mechanisms in the kidney has not been well studied neither in humans nor in SHR. As such, this study was undertaken to see the effect of enalapril, a widely used ACEi class antihypertensive drug, on the control of hypertension and the role of oxidative stress and antioxidant defence mechanisms in hypertension, as the subsequent renal damage progresses in SHR and L-NAME administered SHR.

## 2. Methods

### 2.1. Animals

Male SHR and Wistar-Kyoto (WKY) rats aged just below 4 weeks, obtained from the Animal Research and Service Centre (ARASC), Health Campus, Universiti Sains Malaysia, Kelantan, Malaysia, were used for the study.

### 2.2. Experimental Protocols

The experimental protocols used in this study were approved by the Animal Ethics and Welfare Committee of Universiti Sains Malaysia, Kelantan, Malaysia.

SHR were divided into 4 different groups of six rats each:

1. SHR (untreated): SHR,
2. SHR treated with enalapril (age: 4 weeks–28 weeks): SHR+E,
3. SHR administered L-NAME (age: 16 weeks–28 weeks): SHR+LN,
4. SHR treated with enalapril (age: 4 weeks–28 weeks) and L-NAME (age: 16 weeks–28 weeks): SHR+E+LN.

Control normotensive WKY rats were similarly divided into 4 groups (*n* = 6/group) and treated in the same manner as SHR groups. Each rat was housed in individual cage in standard controlled environment: room temperature of 25–27°C under 12-hour-light and 12-hour-dark cycle (lights on 0700–1900 hours). The animals were fed with standard commercial rat food and water* ad libitum*.

### 2.3. Enalapril and L-NAME Administration

After acclimatization of the rats in the cages, the average daily water intake of rats was determined. Both enalapril (Ranbaxy, Malaysia) and L-NAME (Sigma Chemicals, USA) were given to rats through their daily drinking water in the following doses: enalapril 30 mg kg^−1^ day^−1^, L-NAME 25 mg kg^−1^ day^−1^. Both dosage formulations were prepared freshly each day by dissolving the compounds in slightly less volume of daily water consumption to ensure their complete dosage intake. The daily water consumption was monitored to ensure the dosage was adhered to. Extra drinking water was provided after the required dosage had been taken. Concentration of both compounds in water was adjusted accordingly to match the age-related increase in body weight of the rats.

### 2.4. Physical Parameter Measurements

Body weight of rats was measured every week using a top pan balance by placing the rat in a small weighed cage. SBP was measured every two weeks in conscious rats during the experimental period by the noninvasive (indirect) blood pressure (NIBP) tail plethysmography method, using an automated cuff inflator-pulse detection system (Model 6R22931, IITC Life Science, USA). An average of three readings was taken for each measurement.

### 2.5. Specimen Collection and Processing

One to two days before 4 weeks, 16 weeks, and 28 weeks of age, the rats were placed in metabolic cages for collection of 24-hour urine. Collected urine was stored at −80°C until analysis. Rats were weighed and sacrificed at the end of 28 weeks. Blood samples were collected in plain tubes, allowed to clot, centrifuged to obtain serum, and then stored at −80°C until analysis. Kidneys were rapidly removed, washed in saline, decapsulated, blot-dried, and weighed. One kidney was cut transversely and one half was used for histopathology examination. Other kidney tissues were used for kidney homogenate preparation.

### 2.6. Histopathology Examination

Routine histopathology procedures were followed whereby kidney sections were fixed with 10% neutral buffered formaldehyde for 2 days, dehydrated, and then embedded in paraffin. Paraffin sections were made at 3 *μ*m and stained with haematoxylin/eosin (HE) for microscopic study to assess any glomerular, tubular, and vascular changes.

### 2.7. Preparation of Kidney Homogenates

A weighed amount of kidney tissue was homogenized to make 10% homogenates (w/v) in ice cold (0–4°C) 0.05 M sodium phosphate buffer pH 7.4, using an ice-chilled glass homogenizing vessel in a homogenizer fitted with Teflon pestle (Glass-Col, USA) at 900 rpm. The homogenates were centrifuged in a refrigerated centrifuge at 1,000 ×g at 4°C for 10 minutes to remove nuclei and debris [27]. The supernatant obtained was used for biochemical assays. TBARS assay was carried out on the day of sacrifice. Homogenates were kept frozen at −80°C until analysis for the other assays.

### 2.8. Biochemical Assays

#### 2.8.1. Total Protein

Protein concentration of urine and kidney homogenates was determined using the Micro TP kit (Wako Pure Chemicals, Japan) according to the method of Watanabe et al. [28]. This method is a pyrogallol dye-binding spectrophotometric assay with bovine serum albumin (BSA) as the standard. To 1 mL of the Micro TP reagent 0.01 mL of sample or BSA standard was added and mixed. The reaction mixtures were left at room temperature for 15 minutes before absorbance was read at wavelength of 600 nm using a spectrophotometer (Ultrospec 1100 Pro, UK). Protein concentration in mg/day (for urine) and mg/L (for kidney homogenates) was calculated using the BSA standard.

#### 2.8.2. Creatinine

Serum and urine creatinine were determined by the kinetic alkaline picrate method using a commercial reagent kit (Randox Laboratories, Crumlin, UK). Creatinine clearance was calculated from these data.

### 2.9. Oxidative Stress Markers

#### 2.9.1. TAS

TAS was assessed according to the method of Koracevic et al. [29]. It is based on the principle that a standardized solution of Fe-EDTA complex reacts with hydrogen peroxide by a Fenton-type reaction, leading to the formation of hydroxyl radicals. These reactive oxygen species degrade benzoate, resulting in the release of TBARS. Antioxidants from the added sample of kidney homogenate cause suppression of the production of TBARS that was proportional to their concentration. This reaction is measured spectrophotometrically at 532 nm and the inhibition of colour development is defined as the TAS. The assay was performed as follows.

10 *μ*L of kidney homogenate was pipetted in a test tube containing 0.49 mL of 100 mM sodium phosphate buffer. This was followed by the addition of 0.5 mL of 10 mM sodium benzoate solution, 0.2 mL of Fe-EDTA mixture, and 0.2 mL of 10 mM H_2_O_2_ solution. Negative control (with phosphate buffer instead of the kidney homogenate) containing similar reagents as in sample test tubes was also prepared. The test tubes were vortexed and incubated at 37°C for 60 minutes. This was followed by the addition of 1 mL of 20% acetic acid and 0.8% (w/v) thiobarbituric acid (TBA). The reaction tubes were incubated at 100°C for 10 minutes. After cooling to room temperature, the absorbance of the mixture was measured spectrophotometrically at 532 nm against distilled water. TAS in the kidney homogenates was calculated using uric acid as standard. TAS was expressed as *μ*mol uric acid equivalent per mg protein.

#### 2.9.2. TBARS

Lipid peroxidation was determined as TBARS according to the method of Chatterjee et al. [30]. MDA, an end product of fatty acid peroxidation, reacts with TBA to form a coloured complex which has maximum absorbance at 532 nm. 1,1,3,3-Tetraethoxypropane (TEP), a form of MDA, was used as standard in this assay. Briefly, 1.5 mL of 20% glacial acetic acid (pH 3.5), 0.2 mL of 8.1% sodium dodecyl sulphate (SDS), 1.5 mL of 0.8% (w/v) thiobarbituric acid (TBA), 0.7 mL of distilled water, and 0.1 mL of kidney homogenate or MDA standard were pipetted into test tubes. The test tubes were vortexed (Stuart, UK) and then kept in a boiling water bath (Memmert, Germany) at 95°C for 60 minutes with a marble on top of each test tube. After cooling, the test tubes were centrifuged at 3000 ×g for 10 minutes. One mL of each supernatant was transferred to cuvette and absorbance was read at 532 nm on a spectrophotometer (Ultrospec 1100 Pro, UK). The concentration of each sample was determined from a standard curve based on its absorbance. TBARS levels were represented as *μ*mol MDA equivalent per mg protein.

### 2.10. Antioxidant Enzymes

#### 2.10.1. SOD

SOD activity was assayed according to the method of Dogan et al. [31]. The oxidation of epinephrine is followed in terms of the production of adrenochrome which exhibits an absorption maximum at 480 nm. SOD removes O_2_^.−^ from reaction mixtures by catalyzing its dismutation to O_2_ and H_2_O_2_ thereby inhibiting autoxidation of epinephrine. Measurement of autoxidation of epinephrine was determined by pipetting 2 mL of 0.08 M sodium bicarbonate buffer solution (pH 10.2) into a cuvette, followed by 0.5 mL of 0.75 mM ethylenediaminetetraacetic acid (EDTA) solution. The reaction was started by adding 0.5 mL of 4.37 mM epinephrine into the cuvette and changes in absorbance were measured at wavelength of 480 nm at 30-second intervals for 6 minutes after adding epinephrine. Measurement of SOD in kidney homogenate was performed by pipetting 1.95 mL of 0.08 M sodium bicarbonate buffer solution (pH 10.2), 0.5 mL of 0.75 mM EDTA solution, and 0.05 mL of kidney homogenate into a cuvette. Changes in absorbance were read at wavelength 480 nm every 30 seconds over a period of 6 minutes after adding 0.5 mL of 4.37 mM epinephrine solution, using a spectrophotometer. SOD activity was expressed as unit per mg protein. One unit (U) of SOD was defined as the amount of enzyme that inhibits the rate of autoxidation of epinephrine by 50%.

#### 2.10.2. CAT

CAT activity was assayed according to the method of Goth [32]. The method is based on the enzyme-catalyzed decomposition of hydrogen peroxide and assay of the remaining hydrogen peroxide. Hydrogen peroxide and molybdate ions formed a yellowish complex which has maximum absorbance at 405 nm. The assay requires 4 reaction tubes: Blank 1, Blank 2, Blank 3, and the sample. For Blank 1, Blank 2, and Blank 3, the reagents were added in sequence. Blank 1 contains 0.5 mL substrate (65 mM hydrogen peroxide in 60 mM sodium-potassium phosphate buffer, pH 7.4), 0.5 mL 32.4 mM ammonium molybdate solution, and 0.1 mL kidney homogenate; Blank 2 contains 0.5 mL substrate, 0.5 mL ammonium molybdate solution, and 0.1 mL sodium-potassium phosphate buffer; Blank 3 contains 0.6 mL sodium-potassium phosphate buffer and 0.5 mL ammonium molybdate. For sample tubes, 0.1 mL kidney homogenate was incubated in 0.5 mL substrate at 37°C for 60 seconds. The enzymatic reaction was stopped with 0.5 mL ammonium molybdate solution and the yellow complex of molybdate and hydrogen peroxide was measured at wavelength of 405 nm against Blank 3. CAT activity was expressed as unit per mg protein. One unit of CAT was defined as the amount of enzyme that catalyzes the decomposition of 1 *μ*mol of hydrogen peroxide per minute.

### 2.11. Statistical Analysis

Data were analyzed by one-way ANOVA with post hoc Tukey test using Statistical Package for the Social Science (SPSS) software version 20. Significant level was set (*P* < 0.05). Data are expressed as mean and standard error mean (mean ± SEM) for six animals in each group.

## 3. Results

### 3.1. Kidney Weight and Kidney to Body Weight Ratio

**Figure 1:**
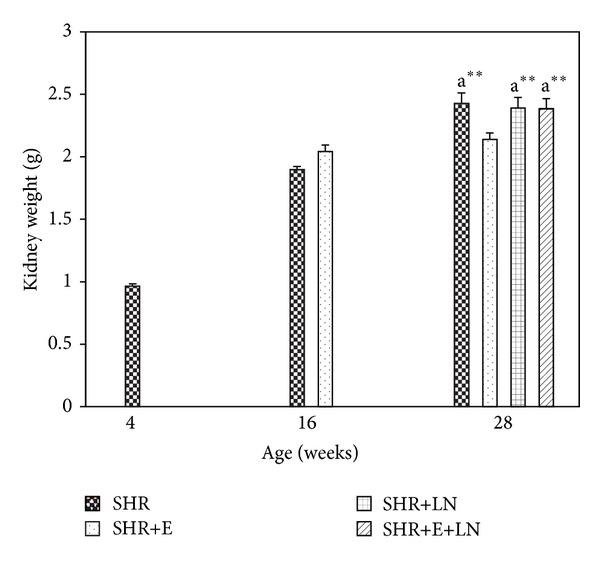
Kidney weight of enalapril treated and untreated SHR and SHR administered L-NAME. a**P < 0.01 SHR+E compared to SHR, SHR+LN, and SHR+E+LN.

**Figure 2:**
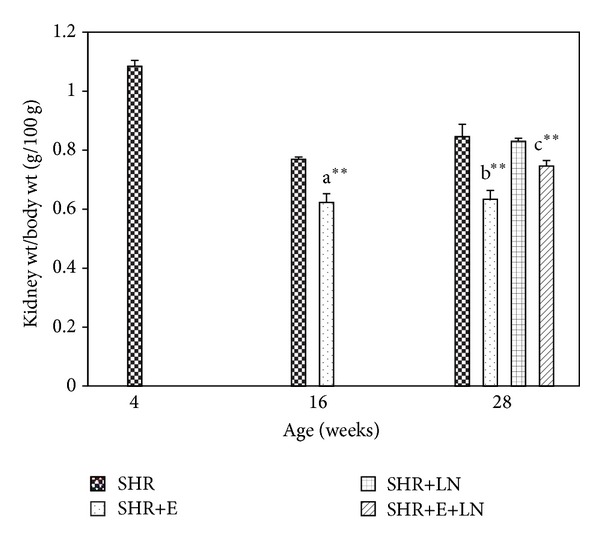
Kidney to body weight ratio of enalapril treated and untreated SHR and SHR administered L-NAME. a**P < 0.01 SHR+E compared to SHR (16 weeks), b**P < 0.01 SHR+E compared to SHR (28 weeks), and c**P < 0.01 SHR+E+LN compared to SHR+LN.

There was no significant difference in the absolute kidney weight of SHR and SHR+E at 16 weeks. However at 28 weeks the absolute kidney weight of SHR, SHR+LN, and SHR+E+LN was significantly increased (*P* < 0.01, a**) when compared to SHR+E. There was no significant difference in absolute kidney weight among SHR, SHR+LN, and SHR+E+LN at 28 weeks (Figure 1). The kidney to body weight ratio for SHR+E was significantly reduced at both 16 weeks and 28 weeks when compared to SHR, SHR+LN, and SHR+E+LN (*P* < 0.01, b**). Kidney to body weight ratio was also significantly reduced in SHR+E+LN at 28 weeks when compared to untreated SHR+LN (*P* < 0.01, c**) (Figure 2).

### 3.2. SBP

**Figure 3:**
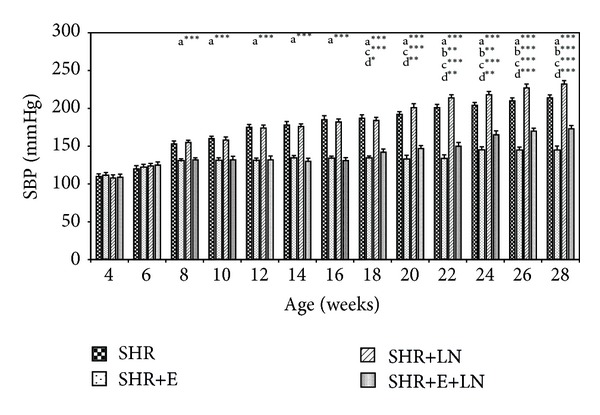
SBP of enalapril treated and untreated SHR and SHR administered L-NAME. a***P < 0.001 SHR compared to SHR+E, b**P < 0.01, b***P < 0.001 SHR+LN compared to SHR, c***P < 0.001 SHR+E+LN compared to SHR+LN, d*P < 0.05, d**P < 0.01, and d***P < 0.001 SHR+E+LN compared to SHR+E. (Note that from week 4 to week 16 the data for the groups SHR+LN and SHR+E+LN are approximately similar to that of SHR and SHR+E rats, respectively, that have not been treated with L-NAME yet.)

The SBP of enalapril treated and untreated SHR and SHR+LN are presented in Figure 3. SBP of SHR treated with enalapril (SHR+E) were significantly lower from the age of 8 weeks until that of 28 weeks when compared to untreated SHR (*P* < 0.001, a***). L-NAME was administered to rats at the age of 16 weeks onwards. After administration of L-NAME, SHR+LN showed significant increase in SBP from week 22 until week 28 compared to SHR (*P* < 0.01 b**; *P* < 0.001, b***). The SHR+LN group treated with enalapril (SHR+E+LN) showed significant decrease compared to untreated SHR+LN (*P* < 0.001, c***) from week 18 until week 28. However the SBP levels of this SHR+E+LN group were still above normal at weeks 22, 24, 26, and 28. When compared to the SHR+E group, the SBP of the SHR+E+LN group showed significant increase from week 18 onwards until week 28 (*P* < 0.05, d*, *P* < 0.01, d**, and *P* < 0.001, d***).

### 3.3. Histopathological Examination

**Figure 4:**
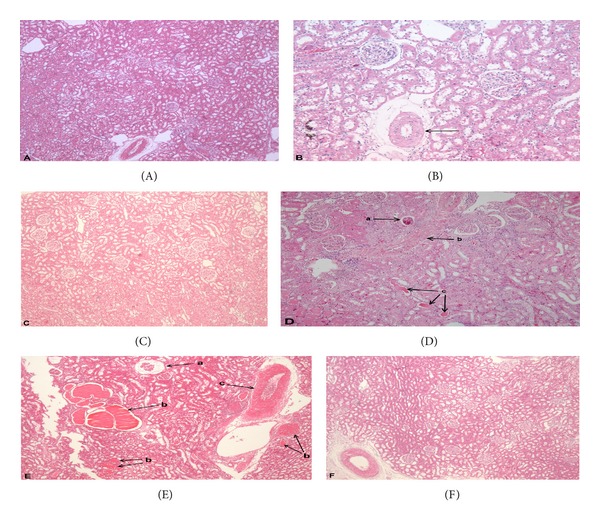
Kidney section HE stains of enalapril treated and untreated SHR and SHR administered L-NAME. (A) SHR at 16 weeks showing no abnormal changes (40x); (B) SHR at 28 weeks showing presence of mild blood vessel changes, medial hypertrophy (100x); (C) SHR+E at 28 weeks showing no abnormal changes (40x); (D) SHR+LN at 28 weeks showing collapsed glomerulus (a), blood vessel hypertrophy (b), and tubular casts (c)(40x); (E) SHR+LN at 28 weeks showing collapsed glomerulus (a), casts (b), and blood vessel hypertrophy (c) (100x); (F) SHR+E+LN at 28 weeks showing no abnormal changes.

Histopathological examination showed no pathological glomerular, tubular, or blood vessel changes in SHR at 4 and 16 weeks. However at 28 weeks, SHR showed some presence of minimal blood vessel medial hypertrophy. SHR+LN at 28 weeks showed significant pathological changes in the glomerulus, tubules, and blood vessels: glomerulosclerosis, shrunken or collapsed glomeruli, increased mesangial cells, presence of inflammatory cells, tubular atrophy and dilatation with casts, and blood vessel hypertrophy. These pathological changes were prevented by enalapril treatment (Figure 4).

### 3.4. Biochemical Parameters

#### 3.4.1. Urinary Protein

**Figure 5:**
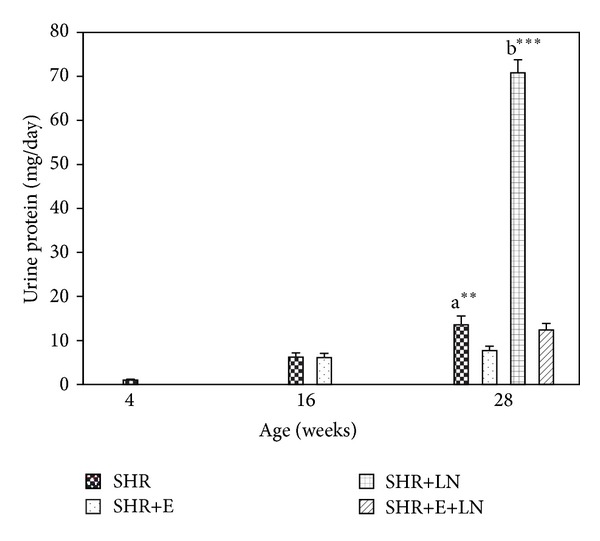
Urine protein levels in enalapril treated and untreated SHR and SHR administered L-NAME. a**P < 0.01 SHR compared to SHR+E; b***P < 0.001 SHR+LN compared to SHR+E+LN.

Figure 5 shows the urinary protein levels in enalapril treated and untreated SHR and SHR+LN. Urinary protein was significantly increased in the untreated SHR group at 28 weeks when compared to the SHR+E group (*P* < 0.01, a**). The greatly increased proteinuria in the SHR+LN group was significantly reduced when treated with enalapril (SHR+E+LN group: *P* < 0.001, b***).

#### 3.4.2. Creatinine Clearance

**Figure 6:**
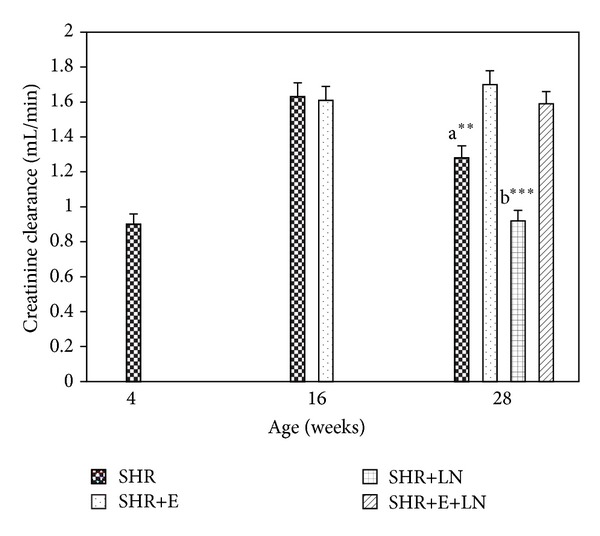
Creatinine clearance levels in enalapril treated and untreated SHR and SHR+LN. a**P < 0.01 SHR compared to SHR+E; b***P < 0.001 SHR+LN compared to SHR+E+LN.

Figure 6 shows the creatinine clearance levels in enalapril treated and untreated SHR and SHR+LN. Creatinine clearance was significantly reduced in the untreated SHR+LN group when compared with the SHR+E+LN group (*P* < 0.001, b***).

#### 3.4.3. TAS

**Figure 7:**
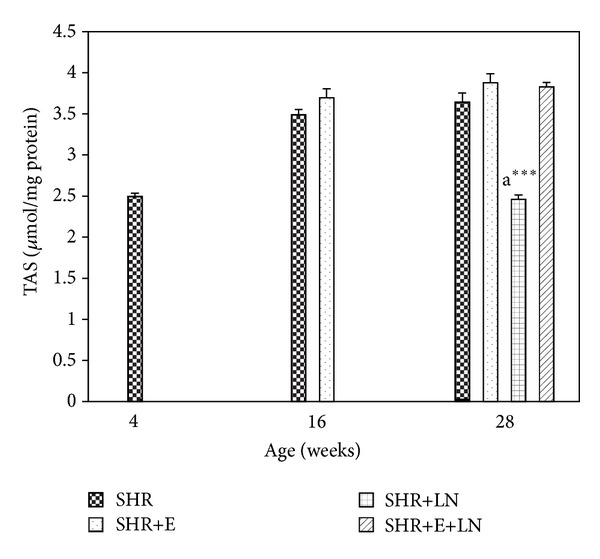
Kidney TAS levels in enalapril treated and untreated SHR and SHR administered L-NAME. a***P < 0.001 SHR+LN compared to SHR, SHR+E, and SHR+E+LN.

Figure 7 represents the kidney TAS levels in enalapril treated and untreated SHR and SHR+LN. There was no significant difference in TAS levels between SHR and SHR+E at 16 and 28 weeks. However the SHR+LN group showed significantly reduced TAS levels at 28 weeks when compared with the other groups (*P* < 0.001, a***).

#### 3.4.4. TBARS

**Figure 8:**
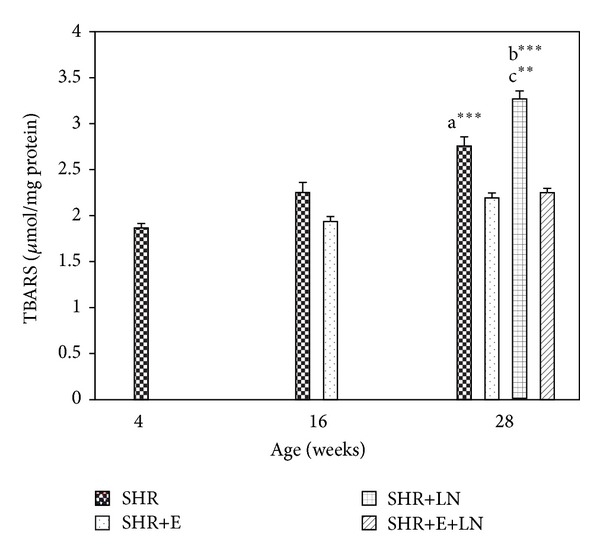
Kidney TBARS levels in enalapril treated and untreated SHR and SHR administered L-NAME. a***P < 0.001 SHR compared to SHR+E, b***P < 0.001 SHR+LN compared to SHR+E and SHR+E+LN, and c**P < 0.01 SHR+LN compared to SHR.

The kidney TBARS levels of enalapril treated and untreated SHR and SHR+LN are shown in Figure 8. There was no significant difference in TBARS levels between SHR and SHR+E at 16 weeks. However at 28 weeks, SHR showed significant increase in TBARS when compared to SHR+E (*P* < 0.001, a***). SHR+LN had the highest TBARS levels at 28 weeks, showing significant increase when compared to all the other groups (*P* < 0.001, b***). Results also showed that enalapril treatment successfully prevented the increase in TBARS levels in both SHR and SHR+LN rat groups at 28 weeks.

#### 3.4.5. SOD

**Figure 9:**
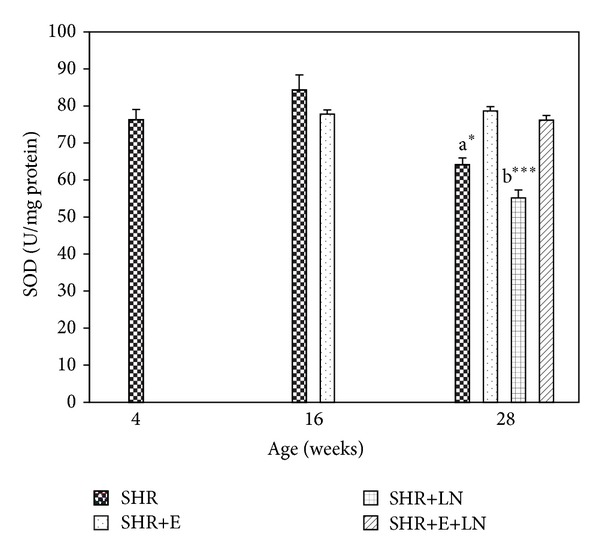
Kidney SOD activity in enalapril treated and untreated SHR and SHR administered L-NAME. a*P < 0.05 SHR compared to SHR+E; b***P < 0.001 SHR+LN compared to SHR, SHR+E, and SHR+E+LN.

There was no significant difference in kidney SOD activity between SHR and SHR+E at 16 weeks. At 28 weeks, SHR showed significant decrease in SOD when compared to SHR+E (*P* < 0.05, a*). SHR+LN had the lowest SOD levels at 28 weeks, showing significant decrease when compared to all the other groups (*P* < 0.001, b***). Results also showed that enalapril treatment successfully enhanced SOD levels in both SHR+E and SHR+E+LN rat groups at 28 weeks (Figure 9).

#### 3.4.6. CAT

**Figure 10:**
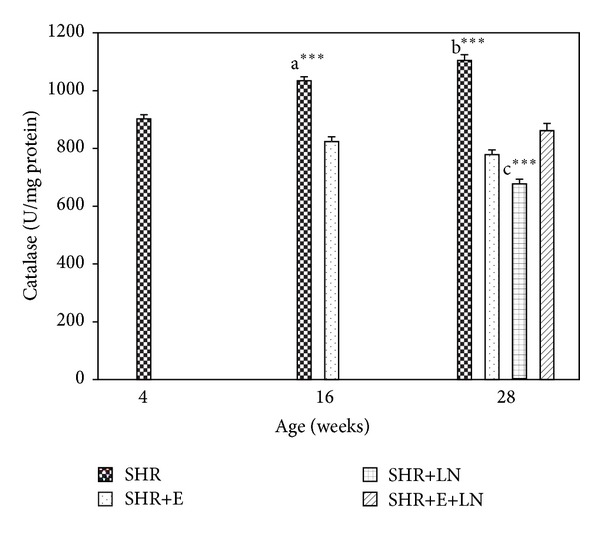
Kidney CAT activity in enalapril treated and untreated SHR and SHR administered L-NAME (16 weeks: a***P < 0.001 SHR compared to SHR+E; 28 weeks: b***P < 0.001 SHR compared to SHR+E and c***P < 0.001 SHR+LN compared to SHR, SHR+E, and SHR+E+LN).

Kidney CAT activity (Figure 10) was significantly increased in SHR at 16 weeks (*P* < 0.001, a***) and 28 weeks (*P* < 0.001, b***) when compared to SHR+E group. CAT activity was the lowest in the SHR+LN group at 28 weeks and significantly reduced when compared to the other groups (*P* < 0.001, c***).

### 3.5. SBP, Biochemical, Oxidative Stress Parameters, and Antioxidant Enzyme Levels in Enalapril Treated and Untreated WKY and WKY+L-NAME

WKY rats showed normal SBP throughout the study period for the WKY and WKY+E groups. L-NAME administration in WKY significantly increased the SBP from 20 weeks onwards (*P* < 0.001) when compared to untreated WKY. Enalapril treatment significantly reduced the SBP in WKY+LN when compared to untreated WKY+LN; however the level was still slightly above normal (data not shown).

Table 1 shows the urinary protein, creatinine clearance, and kidney oxidative stress parameters and antioxidant enzymes SOD and CAT of treated and untreated normotensive WKY and WKY+LN rats. At 28 weeks, WKY+LN had significantly reduced TAS levels when compared to the other groups (a***P < 0.001). Similarly, SOD was significantly reduced (a**P < 0.01) and TBARS significantly increased (a***P < 0.001) in the WKY+LN group at 28 weeks when compared to the other groups. No significant difference was seen in CAT activity between the different groups at 16 and 28 weeks. At 28 weeks, urinary protein was significantly increased (a**P < 0.01) and creatinine clearance significantly decreased (a**P < 0.01) in the WKY+LN group when compared to the other groups.

## 4. Discussion 

This study utilized the SHR to look into the relationship between oxidative stress, kidney damage, and blood pressure lowering effect of enalapril in hypertension in a time-course manner, as this model has been shown to be excellent for the study of hypertension [33]. The SHR+L-NAME model was incorporated into the study so as to hasten the kidney damage and thereby shorten the study period. Zhou and Frohlich [23] showed the suitability of this model whereby they started the L-NAME inhibition on 17-week-old SHR, producing clear nephropathy in 3 weeks. Similarly, in our study, L-NAME inhibition was commenced at around the same age, that is, at 16 weeks, and continued for 12 weeks until 28 weeks so as to ensure that significant and extensive nephropathy occurs, which was confirmed by our histopathology, proteinuria, and creatinine clearance results. L-NAME inhibition was not started at an earlier age as enhancing hypertension rapidly at a younger age might affect the survival rate of the rats. Overall the study time points of 4 weeks, 16 weeks, and 28 weeks were selected so as to observe the changes from prehypertension to established hypertension and finally hypertensive kidney damage periods. The 16- and 28-week study time points were also selected as our previous research showed that this age period had greater increase in blood pressure and antioxidant changes [34]. Enalapril, a widely used ACEi class of antihypertensive drugs, was used for this study as it has been said to have renoprotective properties but the exact mechanism for this is not known [35]. The enalapril and L-NAME doses used in this study are similar to what has been used by other researchers [36, 37].

Results obtained showed that SBP of untreated SHR was already elevated to hypertensive levels at 8 weeks of age. This is in agreement with other researchers who noted hypertension in SHR at around this age [38, 39]. This elevated blood pressure increased progressively with a sharp increase occurring between the ages of 16 and 28 weeks. Chronic inhibition of nitric oxide synthase with L-NAME to SHR, initiated at 16 weeks, caused significant increase in SBP from 20 weeks onwards when compared to untreated SHR. SBP exceeded more than 200 mmHg after 4 weeks of administration and reached more than 220 mmHg at the end of the experimental period at 28 weeks. This confirmed the effect of L-NAME on SBP as obtained by other researchers [24–26]. Enalapril administration to SHR succeeded in lowering the SBP within normal limits. However, for the SHR+LN group, enalapril administration did not effectively reduce the SBP to normal whereby the values were about 155 mmHg at 24 to 28 weeks (Figure 3). The blood pressure of enalapril treated and untreated WKY rats was normal and relatively unchanged throughout the study. However the WKY+LN group showed elevated SBP, almost similar to the SHR+LN group (data not shown). Here, again, enalapril administration did not effectively reduce the SBP to normal. The reason for both these situations could be because the enalapril dose used was insufficient to overcome the inhibition effects caused by L-NAME.

SHR showed significantly lower body weight than age-matched WKY from 10 weeks onwards (data not shown). This could be due to various factors including metabolic changes associated with hypertension, stress, and poorer appetite. The absolute kidney weight of untreated SHR, SHR+LN, and SHR+E+LN at 28 weeks was significantly higher than SHR+E. The kidney to body weight ratio was also increased in a similar pattern (Figures 1 and 2). This is probably due to hypertrophy of various structures in the kidney brought about by hypertension which causes the kidney weight to increase as well as the lower body weight of these groups which results in a reduced kidney to body weight ratio. Similar findings were reported by researchers experimenting on different animal models of hypertension [40, 41].

The histopathology results of this study confirmed the effect of L-NAME in producing kidney damage as clear pathological changes were seen in the glomerulus, tubules, and blood vessels at 28 weeks (Figures 4(D)-4(E)). Besides this, urine protein was markedly increased and the creatinine clearance was greatly reduced. Enalapril treatment managed to prevent this damage, confirming its renoprotective effect through blood pressure lowering as mentioned by other researchers [35].

Oxidative stress has been implicated in the pathogenesis and progression of hypertension with some studies suggesting it is the cause while others suggest it is a consequence of hypertension [4, 5, 42, 43]. Results from this study showed that TAS levels are significantly reduced at 28 weeks in SHR+LN rats when compared to the other groups. Also, TBARS levels are significantly raised in SHR and SHR+LN rats during the same time period. These findings indicate the presence of oxidative stress in the kidneys of these groups. The SBP of these rat groups during this time period was also very high, exceeding 200 mmHg, indicating a strong relationship with oxidative stress. Enalapril treatment, besides reducing the SBP, also managed to prevent this oxidative stress by reducing the TBARS levels as well as enhancing the TAS levels (Figures 5 and 6). SOD levels at 28 weeks were significantly reduced in the SHR and SHR+LN rats. These levels were restored to earlier 4-week and 16-week levels when enalapril was administered (Figure 7). CAT activity in the SHR group was significantly raised at 16 weeks and 28 weeks when compared to the other groups. It is possible that this overexpressed CAT activity during this time might be a compensatory mechanism to protect the kidney from the deleterious effects of free radicals involved in causing oxidative stress. The CAT activity in the SHR+LN group was significantly reduced at 28 weeks when compared to the other groups. This could be due to some unknown mechanism in the oxidative stress process that has affected its activity. Enalapril treatment managed to normalise the CAT activity to earlier levels (Figure 8). All these findings clearly indicate that enalapril has antioxidative properties. This study also supports the view that enalapril has renoprotective properties which might be conferred through the reduction or elimination of oxidative stress in the kidney as has been shown for other antioxidants [44].

## 5. Conclusion

This study showed that enalapril, in addition to blood pressure lowering properties, also has beneficial effect in reducing oxidative stress in the kidneys.

## Figures and Tables

**Table 1:** Effect of enalapril treatment on urinary protein, creatinine clearance, kidney TAS, TBARS, SOD, and CAT levels in treated and untreated WKY and WKY administered L-NAME rats.

Parameters	Groups				
	Age	WKY	WKY+E	WKY+LN	WKY+E+LN
TAS (umol/mg protein)	4 weeks	2.52 ± 0.03	—	—	—
	16 weeks	3.21 ± 0.06	3.34 ± 0.06	—	—
	28 weeks	3.47 ± 0.04	3.48 ± 0.05	2.67 ± 0.12^a∗∗∗^	3.68 ± 0.06

TBARS (umol/mg protein)	4 weeks	1.87 ± 0.05	—	—	—
	16 weeks	1.96 ± 0.11	1.94 ± 0.16	—	—
	28 weeks	2.28 ± 0.11	2.23 ± 0.13	3.05 ± 0.12^a∗∗∗^	2.15 ± 0.06

SOD (U/mg protein)	4 weeks	82.50 ± 5.8	—	—	—
	16 weeks	78.50 ± 3.94	82.33 ± 2.53	—	—
	28 weeks	79.50 ± 3.10	78.83 ± 2.20	61.33 ± 1.17^a∗∗^	74.83 ± 0.95

CAT (U/mg protein)	4 weeks	857 ± 21	—	—	—
	16 weeks	781 ± 14	706 ± 7	—	—
	28 weeks	768 ± 12	698 ± 15	725 ± 22	699 ± 16

Urinary protein (mg/day)	4 weeks	0.98 ± 0.22	—	—	—
	16 weeks	5.16 ± 0.41	6.08 ± 0.42	—	—
	28 weeks	5.28 ± 0.34	5.95 ± 0.43	9.19 ± 0.39^a∗∗^	5.80 ± 0.30

Creatinine clearance (mL/min)	4 weeks	0.59 ± 0.05	—	—	—
	16 weeks	1.69± 0.06	1.67 ± 0.07	—	—
	28 weeks	1.79± 0.08	1.77 ± 0.08	1.47 ± 0.03^a∗∗^	1.76 ± 0.07

Values are expressed as mean ± SEM (*n* = 6 per group).

WKY: WKY with no treatment, WKY+E : WKY+enalapril, WKY+LN : WKY+L-NAME, and WKY+E+LN : WKY+enalapril+L-NAME.

^a∗∗^ *P* < 0.01 and ^a∗∗∗^ *P* < 0.001 WKY+LN compared to WKY, WKY+E, and WKY+E+LN.

## References

[1] Chobanian AV, Bakris GL, Black HR (2003). The Seventh report of the joint national committee on prevention, detection, evaluation, and treatment of high blood pressure: the JNC 7 report. *The Journal of the American Medical Association*.

[2] World Health Statistics 2012. http://www.who.int/gho/publications/world_health_statistics/2012/en.

[3] Swales JD (1994). *Textbook of Hypertension*.

[4] Touyz RM (2000). Oxidative stress and vascular damage in hypertension.. *Current hypertension reports*.

[5] Wilcox CS (2002). Reactive oxygen species: roles in blood pressure and kidney function. *Current Hypertension Reports*.

[6] McCord JM (1993). Human disease, free radicals, and the oxidant/antioxidant balance. *Clinical Biochemistry*.

[7] Lunec J (1990). Free radicals: Their involvement in disease processes. *Annals of Clinical Biochemistry*.

[8] Halliwell B, Gutteridge JMC, Cross CE (1992). Free radicals, antioxidants, and human disease: where are we now?. *Journal of Laboratory and Clinical Medicine*.

[9] Manning RD, Tian N, Meng S (2005). Oxidative stress and antioxidant treatment in hypertension and the associated renal damage. *The American Journal of Nephrology*.

[10] Tse WY, Maxwell SRJ, Thomason H (1994). Antioxidant status in controlled and uncontrolled hypertension and its relationship to endothelial damage. *Journal of Human Hypertension*.

[11] Russo C, Olivieri O, Girelli D (1998). Anti-oxidant status and lipid peroxidation in patients with essential hypertension. *Journal of Hypertension*.

[12] Minuz P, Patrignani P, Gaino S (2002). Increased oxidative stress and platelet activation in patients with hypertension and renovascular disease. *Circulation*.

[13] Lacy F, Kailasam MT, O'Connor DT, Schmid-Schönbein GW, Parmer RJ (2000). Plasma hydrogen peroxide production in human essential hypertension: role of heredity, gender, and ethnicity. *Hypertension*.

[14] Laverman GD, Remuzzi G, Ruggenenti P (2004). Ace inhibition versus angiotensin receptor blockade : which is better for renal and cardiovascular protection. *Journal of the American Society of Nephrology*.

[15] Berl T (2004). Angiotensin-converting enzyme inhibitors versus AT1 antagonist in cardiovascular and renal protection : the case for AT1 receptor antagonist. *Journal of the American Society of Nephrology*.

[16] Pereira LMM, Almeida JR, Mandarim-de-Lacerda CA (2004). Kidney adaptation in nitric oxide-deficient Wistar and spontaneously hypertensive rats. *Life Sciences*.

[17] Boffa J, Lu Y, Placier S, Stefanski A, Dussaule J, Chatziantoniou C (2003). Regression of renal vascular and glomerular fibrosis: role of angiotensin II receptor antagonism and matrix metalloproteinases. *Journal of the American Society of Nephrology*.

[18] Tong Mak I, Boehme P, Weglicki WB (1992). Antioxidant effects of calcium channel blockers against free radical injury in endothelial cells: correlation of protection with preservation of glutathione levels. *Circulation Research*.

[19] Wiemer G, Linz W, Hatrik S, Schölkens BA, Malinski T (1997). Angiotensin-converting enzyme inhibition alters nitric oxide and superoxide release in normotensive and hypertensive rats. *Hypertension*.

[20] Rajagopalan S, Kurz S, Munzel T (1996). Angiotensin II-mediated hypertension in the rat increases vascular superoxide production via membrane NADH/NADPH oxidase activation. Contribution to alterations of vasomotor tone. *Journal of Clinical Investigation*.

[21] Bayorh MA, Ganafa AA, Socci RR, Eatman D, Silvestrov N, Abukhalaf IK (2003). Effect of losartan on oxidative stress-induced hypertension in Sprague-Dawley rats. *American Journal of Hypertension*.

[22] Mantle D, Patel VB, Why HJF (2000). Effects of lisinopril and amlodipine on antioxidant status in experimental hypertension. *Clinica Chimica Acta*.

[23] Zhou X, Frohlich ED (2007). Analogy of cardiac and renal complications in essential hypertension and aged SHR or L-NAME/SHR. *Medicinal Chemistry*.

[24] Baylis C, Mitruka B, Deng A (1992). Chronic blockade of nitric oxide synthesis in the rat produces systemic hypertension and glomerular damage. *The Journal of Clinical Investigation*.

[25] Wessels J, Peake P, Pussell BA, Macdonald GJ (1997). Nitric oxide synthase inhibition in a spontaneously hypertensive rat model of diabetic nephropathy. *Clinical and Experimental Pharmacology and Physiology*.

[26] Gerová M (2000). Nitric oxide-compromised hypertension: facts and enigmas. *Physiological Research*.

[27] Scott RB, Reddy KS, Husain K, Schlorff EC, Rybak LP, Somani SM (2000). Dose response of ethanol on antioxidant defense system of liver, lung, and kidney in rat. *Pathophysiology*.

[28] Watanabe N, Kamei S, Ohkubo A (1986). Urinary protein as measured with a pyrogallol red-molybdate complex, manually and in a Hitachi 726 automated analyzer. *Clinical Chemistry*.

[29] Koracevic D, Koracevic G, Djordjevic V, Andrejevic S, Cosic V (2001). Method for the measurement of antioxidant activity in human fluids. *Journal of Clinical Pathology*.

[30] Chatterjee PK, Cuzzocrea S, Brown PAJ (2000). Tempol, a membrane-permeable radical scavenger, reduces oxidant stress-mediated renal dysfunction and injury in the rat. *Kidney International*.

[31] Dogan P, Tanrikulu G, Soyuer U, Kose K (1994). Oxidative enzymes of polymorphonuclear leucocytes and plasma fibrinogen, ceruloplasmin, and copper levels in Behcet's disease. *Clinical Biochemistry*.

[32] Goth L (1991). A simple method for determination of serum catalase activity and revision of reference range. *Clinica Chimica Acta*.

[33] Pinto YM, Paul M, Ganten D (1998). Lessons from rat models of hypertension: from Goldblatt to genetic engineering. *Cardiovascular Research*.

[34] Chandran G, Sirajudeen KNS, Tee CW, Nadiger HA (2005). Time course study on oxidative stress in kidney of spontaneously hypertensive rat. *The Malaysian Journal of Medical Sciences*.

[35] Rugale C, Cordaillat M, Mimran A, Jover B (2005). Prevention and reversal by enalapril of target organ damage in angiotensin II hypertension. *Journal of the Renin-Angiotensin-Aldosterone System*.

[36] Dukacz SAW, Feng M, Yang L, Lee RMKW, Kline RL (2001). Abnormal renal medullary response to angiotensin II in SHR is corrected by long-term enalapril treatment. *American Journal of Physiology-Regulatory Integrative and Comparative Physiology*.

[37] Fujihara CK, Malheiros DMAC, Noronha IDL, de Nucci G, Zatz R (2001). Mycophenolate mofetil reduces renal injury in the chronic nitric oxide synthase inhibition model. *Hypertension*.

[38] Dickhout JG, Lee RM (1998). Blood pressure and heart rate development in young spontaneously hypertensive rats. *The American Journal of Physiology—Heart and Circulatory Physiology*.

[39] Lee SK, Arunkumar S, Sirajudeen KNS, Singh HJ (2010). Glutathione system in young spontaneously hypertensive rats. *Journal of Physiology and Biochemistry*.

[40] Oyekan AO, Mcaward K, Conetta J, Rosenfeld L, Mcgiff JC (1999). Endothelin-1 and CYP450 arachidonate metabolites interact to promote tissue injury in DOCA-salt hypertension. *The American Journal of Physiology—Regulatory Integrative and Comparative Physiology*.

[41] Laakso J, Mervaala E, Himberg J (1998). Increased kidney xanthine oxidoreductase activity in salt-induced experimental hypertension. *Hypertension*.

[42] Nosratola DV (2008). Causal link between oxidative stress, inflammation and hypertension. *Iranian Journal of Kidney Diseases*.

[43] Khanna HD, Sinha MK, Khanna S, Tandon R (2008). Oxidative stress in hypertension: association with antihypertensive treatment. *Indian Journal of Physiology and Pharmacology*.

[44] Tian N, Thrasher KD, Gundy PD, Hughson MD, Manning RD (2005). Antioxidant treatment prevents renal damage and dysfunction and reduces arterial pressure in salt-sensitive hypertension. *Hypertension*.

